# Effect of hydroponic green herbage on the productive qualities of parent flock geese

**DOI:** 10.14202/vetworld.2021.841-846

**Published:** 2021-04-08

**Authors:** Danis Khaziev, Rinat Gadiev, Chulpan Yusupova, Marina Kazanina, Svetlana Kopylova

**Affiliations:** 1 Department of Beekeeping, Private Zootechny and Breeding of Animals, Federal State Budgetary Education Institution of Higher Education “Bashkir State Agrarian University,” Ufa, Russia; 2 Laboratory for Genomic Research and Animal Breeding, Federal State Budgetary Scientific Institution “Ural Federal Agrarian Scientific Research Centre, Ural Branch of the Russian Academy of Sciences,” Yekaterinburg, Russia; 3 Department of Morphology, Pathology, Pharmacy and Non-Communicable Diseases, Federal State Budgetary Education Institution of Higher Education “Bashkir State Agrarian University,” Ufa, Russia

**Keywords:** economic efficiency, green food, hydroponics, parent flock geese, productive qualities, sprouted green herbage

## Abstract

**Background and Aim:**

Green food is the natural diet for livestock and poultry. Therefore, production of green food in sufficient quantities to meet the current demand has emerged as an urgent problem today. The use of natural laylands results in green food shortage, which, in turn, necessitates the application of various methods of artificial production of green herbage. One of these methods is hydroponic cultivation of green grass as animal feed. Hence, this study was conducted to investigate the productive and reproductive qualities of geese of the parent herd.

**Materials and Methods:**

Complex scientific analysis was conducted to explore the effect of hydroponic green herbage used at various dosages (20%, 25%, 30%, and 35% of total diet weight) on the realization of the reproductive qualities of parent flock geese. The methodological framework of this research is the efforts of various foreign and domestic scientists on the topic under study. This research was conducted using generally accepted methods (i.e., experiment, comparison, analysis, and generalization), along with special methods (zootechnical, physiological, biological, hematological, morphological, statistical, and economic).

**Results:**

The optimal dosage of hydroponic green herbage for geese diet was established, which constituted 25-30% of the total diet weight and increased the poultry population survival rate by 2.0%, egg production rate by 3.8%, and the hatching egg yield by 4.9%. The carotenoid content in egg yolk ranged from 1.62 to 3.50 μg. The content of Vitamins A and B_2_ was higher by 3.19 and 2.32 μg, respectively, compared to that in the control. The production profitability level increased by 9.6%.

**Conclusion:**

By introducing 25-30% of hydroponic greens from the weight of the diet, it is possible to increase the safety of livestock by 2%, the yield of hatching eggs by 4.9%, egg production by 1.46-1.11 μg.

## Introduction

One of the major problems in poultry farms is to increase productivity through more efficient use of nutrients while reducing production costs. The problem of shortage of feed has become acute in recent years. Therefore, several poultry farms must consider various methods to organize good poultry fattening. Hydroponics is one of such methods used to improve the efficiency of feed use. Hydroponics is a type of horticulture and a subset of hydroculture, which is a method of growing plants, usually crops, without soil using less water at the required temperature and humidity in an ecologically controlled environment. Several animal breeders have switched from traditional feed production methods to the hydroponic method because it is much easier and faster to produce nutritious green herbage using hydroponics [1].

Armanda *et al*. [2] reported on the cultivation of green herbage using the hydroponic method as one of the innovative methods of farming. They indicated that water consumption was 90-95% lower in the hydroponic method than in the traditional agricultural methods. Moreover, the use of pesticides, fungicides, insecticides, and artificial growth promoters is minimized or even not used at all. A combination of hydroponic systems on industrial roofs, gardens, and open spaces can increase the production and demand for vegetables at lower production costs [3-5]. The production of hydroponic feed requires approximately 2-3% of the water used in the field to produce the same amount of feed [6].

Jemimah *et al*. [1] mentioned the following advantages of hydroponic feed cultivation technology: Reduced plant growth time, which takes 8 days compared with traditional conditions where growing takes 45 days; high nutritional value, which implies that hydroponic products contain more raw protein than traditional green products; use of much less land area compared with field conditions; and minimal use of equipment and fuel for repairing, growing, harvesting, transporting, and storing produced products. In addition, hydroponic feed production requires minimal human effort and time. Considering all the positive aspects of this cultivation system of green herbage, it must be noted that the hydroponic method of feed production contributes to the sustainable development of both a separate branch of animal husbandry and agriculture as a whole. This approach increases the profitability of livestock products and helps breeders reduce production costs [7].

The significance of the present study is that the quality of young animals and the productive qualities of geese are influenced by numerous factors. However, the influence of hydroponic greenery on the manifestation of their productive and reproductive qualities has been poorly investigated.

The aim of this study was to investigate the productive and reproductive qualities of geese of the parent herd when including various doses of hydroponic greens in their diet instead of barley and grass flour.

## Materials and Methods

### Ethical approval

This study was conducted according to the regulations for Research in Animal Health and samples were collected as per standard collection methods without causing any harm or stress to the birds.

### Study period and location

The study was conducted for 130 days (February-June 2019) in the conditions of LLC “Bashkir Bird”, Blagovarsky district of the Republic of Bashkortostan.

### Research object and materials

Five groups of parent flock geese of the white Hungarian breed were formed to conduct this research. Each group consisted of 50 birds. Birds of the 2^nd^ year of use with a live weight of 5.8-6.3 kg in male geese and 5.3-5.8 kg in female geese were examined. The amount of metabolic energy in the diet per 100 g is 1.072 Mj (255 kcal). The feeding frequency is 3 times a day. The control group consumed compound feed without hydroponic green herbage. Birds in the experimental Groups 1, 2, 3, and 4 received 20%, 25%, 30%, and 35% of the hydroponic green feed in their diet. The study was conducted for 130 days in the conditions of LLC “Bashkir bird” of Blagovarsky district of the Republic of Bashkortostan.

### Research conditions

According to the recommendations of the All-Russian Scientific Research Poultry Institute (Sergiev Posad city), equal housing and feeding conditions were provided for the poultry population in this study. The birds were kept in strawed pens.

### Statistical analysis

Six birds from the experimental group were used to evaluate the digestibility of dietary nutrients in their body by conducting physiological and balance tests using the All-Russian Scientific Research Poultry Institute method (Sergiev Posad, Russia) All the experimentally obtained data were processed by the variation statistics method using the Microsoft Excel software 2003 (Microsoft, USA) on a personal computer. Student’s t-test was used to determine statistical significance at the following three levels of probability: p<0.05, p<0.01, and p<0.001.

## Results

An important indicator reflecting the results of economic activity is the survival rate of animals, which is determined using feeding adequacy, provision of the body with all the required nutrients, and environmental factors.

**Figure-1:**
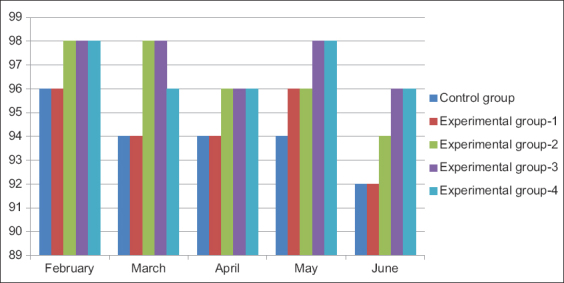
Adult stock survival rate (%).

The introduction of hydroponic green herbage in the geese diet significantly increased the survival rate of poultry population (Figure-1). High safety indicators were observed in all experimental groups, even at the beginning of the productive period. In experimental groups 2, 3, and 4, in which geese received 25-35% of hydroponic green herbage instead of grass meal and barley, the survival rate was 94.0%, which was 2.0% higher than that in the control group. Furthermore, the introduction of hydroponic green herbage at a dose of 25-35% of the diet weight increased the survival rate in all the experimental groups during the entire study period.

The high survival rate can be explained by the excellent provision of poultry with vitamins and minerals, which are abundant in hydroponic green herbage. Therefore, the use of hydroponic green herbage in the diet instead of grass meal and barley helps in increasing the survival rate of the parent flock geese. The most optimal dosage was 25-35% of the total diet weight.

**Figure-2:**
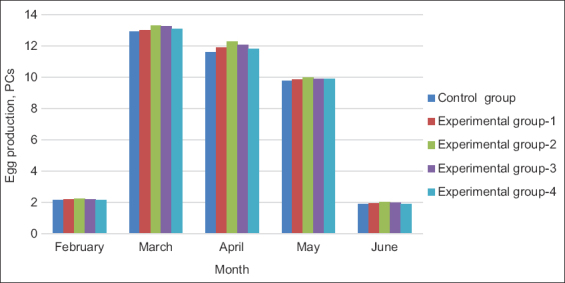
Geese egg production (PCs).

Providing a farm with high-quality hatching eggs for goose meat production throughout the year is an important task of the parent flock. The egg production traits for all months of the productive period are shown in Figure-2. During the productive period of 40 weeks, high egg production was observed in geese in experimental Group 2, which was 3.8% higher than that in the control group. The Figure-2 shows that the introduction of hydroponic green herbage into the diet contributed to an increase in the egg production of egg-laying birds. However, the high fiber content in the diet may slightly reduce the poultry egg production, which was observed in experimental Group 4, in which the amount of green feed was 35%.

The number of obtained eggs is as essential for egg production as egg quality. The results of this study did not reveal any significant effect of hydroponic green herbage on the shell thickness of egg. However, a slight increase in shell thickness was observed in the experimental groups, which could be attributed to the increased provision of poultry with minerals contained in hydroponic green herbage.

The indicators of vitamin content for hatching eggs confirmed that the parent flock geese diets are sufficiently provided with vitamins. In fact, the amount of Vitamins B, A, and C in hydroponic green herbage was comparable to that in fresh carrots and lettuce grown on soil. In addition, hydroponic green herbage is rich in major nutrient elements required for the body [8-10].

In geese that received hydroponic green herbage, the content of vitamins in their eggs increased, which led to an increase in the content of carotenoids in the yolk from 1.62 to 3.50 μg compared to that in the control group. Moreover, by increasing the proportion of hydroponic green herbage in the diet, it is possible to enrich the egg with Vitamins A and B. Therefore, the content of Vitamins A and B in experimental Group 4 was 3.65% and 3.15% higher than that in the control group, respectively.

**Figure-3:**
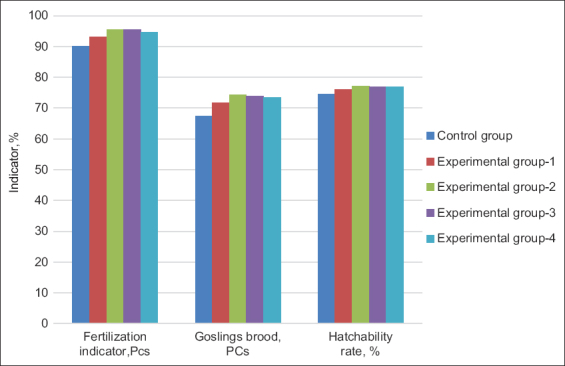
Eggs hatching indicators (%).

The data on the egg incubation qualities are schematically depicted in Figure-3. Fertilization and gosling brood were confirmed to be high indicators in experimental Groups 2 and 3 compared with the same indicators in the control group. However, in experimental Group 3, there was a slight decrease in the hatchability rate, based on which it can be concluded that the introduction of 25% of hydroponic green herbage to the diet is the most productive.

For feed quality assessment, it is essential to understand the extent to which the feed nutrients are used and how well the bird’s body digests them. Physiological experiments were performed during the research to evaluate the digestibility of geese feed nutrients to determine the optimal dose of hydroponic green feed to be introduced instead of barley and grass meal. Balance experiments showed that the introduction of green herbage grown in hydroponic conditions into geese diet has a positive effect on digestibility (Table-1).

**Table-1:** Use of feed nutrients in the diet (%).

Indicator	Group				

	Control group	Experimental group 1	Experimental group 2	Experimental group 3	Experimental group 4
Digestibility (%)					
Protein	78.3±0.38	78.8±0.48	79.5±0.46*	79.3±0.42	78.6±0.42
Fat	55.8±0.18	56.1±0.08	56.4±0.21*	56.2±0.19	55.9±0.19
Fiber	53.1±0.16	52.9±0.24	52.7±0.26*	52.4±0.23*	52.6±023*
USE of nitrogen	46.7±0.41	47.3±0.46	47.9±0.39*	47.7±0.35*	47.3±0.35

Differences from the control are significant:

* p<0.05;

**p<0.01; ***p<0.001

Based on scientific analysis, it was found that in the experimental groups, the digestibility of feed protein was 0.5-1.2% higher than that in the control group. Experimental Group 2, in which the geese diet included 25% of hydroponic green herbage, showed the highest protein indicators, comprising 79.5%. A similar trend was observed for the fat digestibility indicator, which increased by 0.6%. Furthermore, in the experimental groups, feed nitrogen fixation increased by 0.6-1.2%, whereas fiber digestibility decreased, which is associated with feed structure.

High indicators of digestibility and use of feed nutrients also positively affected feed costs per unit production, which decreased by 1.5-4.3% in the experimental groups. Compared with the previous high indicators, the feed cost per unit production in experimental Group 2 was the lowest and resulted in 16.1 kg. In our opinion, the positive effect of feeding geese with hydroponic green herbage can be explained by its stimulating effect on the processes of digestion and metabolism. Therefore, by replacing grass meal and barley in the poultry diet with green feed grown in hydroponic conditions, we can increase the efficiency of feed use, which ultimately leads to a favorable economic situation.

A production inspection was conducted to calculate economic indicators and obtain economic efficiency from hydroponic green herbage added to the parent flock geese diet. The results of this calculation are presented in Table-2. It was observed that due to high productivity, the share of brood and hatching egg yield as well as the cost of daily young geese in the new variant was 3.3 rubles lower (i.e., 8.2%) than that in the basic variant and comprised 40.0 rubles. Hence, the profit from sales increased by 87,021 rubles, and the profitability of production was 25.1%, whereas in the basic variant, it was 15.5%.

**Table-2:** Effectiveness of the hydroponic green herbage use in the parent flock geese diet.

Indicator	Variant	

	Basic	New
Total flock	1000	1000
Including She-geese	750	750
He-geese	250	250
Egg production per average egg-laying bird (PCs)	38.1	39.6
Goslings brood (%)	67.2	73.3
Hatching eggs yield (%)	94.9	96.1
The total number of eggs (PCs)	27118	28542
The number of daily goslings (head)	18223	20921
The total feed consumption (kg)	37453	38337
The total feed cost (RUB)	151647.2	141770.2
The cost of the diet including hydroponic green herbage (RUB/t)	4049.97	3698.9
Cost of 1 head of daily young stock (RUB)	43.3	40.0
Total cost for egg production (RUB)	788565.4	836444.3
Total revenue (RUB)	911154	1046053
Profit (RUB)	122588	209609
Profitability level (%)	15.5	25.1

## Discussion

The choice of hydroponic feed for its further production depends on geographical and agroclimatic conditions and seed availability. The grain selected for hydroponics production must be clean, safe for animal health, undamaged or free from insect infestation, free from chemicals, viable, and of good quality, that is, available for producing the best quality biomass.

Galin *et al*. [8] suggested that for the production of sprouted greens, the grain must be pre-steeped. Steeping helps absorb water and release materials for seed growth and development rapidly. It has been shown that a nutrient solution can sometimes be replaced with tap water for hydroponic feed production. Moreover, the seeding rate also affects the yield. The seeding rate proposed by Galin *et al* [8] was 7.6 kg/m^2^.

According to Al-Karaki and Al-Hashimi [6], barley is considered to be the best raw material for the production of hydroponic green feed as it requires the least amount of water, and the seeds of this crop are available on the market, and they are inexpensive compared with other crop seeds, which reduces the cost of hydroponic feed production. The results of this study also confirmed this fact.

Brown *et al*. [11] confirmed the positive effect of hydroponic green herbage when used in the diet of pigs. They recorded a high nutritional value and digestibility of sprouted feed and considered it as a possible alternative to traditional green feed. The use of hydroponic corn feed in the diet of weaned pigs improved their productivity and nutrient digestibility. Hence, there is abundant potential for the development of hydroponic feed production technology in pig farming.

Khaziev [12] investigated the effect of hydroponic feed on the productive and reproductive qualities of parent flock ducks. They experimentally established that the introduction of hydroponic green herbage in the diet of ducks increased their survival rate by up to 98%.

Hydroponic green feed, being rich in vitamins, improves the qualities of hatching duck eggs. Moreover, the content of carotenoids in hatching eggs is related to the feed. According to the chemical analysis of eggs in the experimental groups, the content of carotenoids in the yolk increased to 27.0 μg when ducks received hydroponic green herbage.

The hydroponic method aims at producing a high-quality, low-cost feed, and this goal was successfully fulfilled. The use of hydroponic green herbage reduces the cost of feeding by approximately 30%, thereby allowing animal breeders to save capital and reallocate resources to other expenses such as health and education. Moreover, better animal feeding improves animal health and thus reduces veterinary costs. This method is useful and relevant. It meets the needs of farmers without damaging the ecosystem due to its minimal impact on the environment [7].

Gericke (1920-1930) developed a technique for growing plants in nutrient solution on a large scale. In 1939, Leitch analyzed a series of experiments using sprouted feed for various livestock and poultry types and reported that sprouted feed is the commercial exploitation of water plant culture processes for livestock feed production. In 1969, the English scientist Woodward attempted to grow plants in various water sources [11]. There is abundant potential for the development of hydroponics technology for feed production. Hydroponic feed can be produced and fed in situations where feed under cultivation cannot be successfully grown. This technology can also be adopted by progressive modern dairy farmers having elite dairy herds for producing hydroponic feed to feed their dairy animals. However, further investigation is required to develop low-cost feed production devices using this technology and locally available materials [8].

## Conclusion

By introducing hydroponic green herbage at dosages of 25-30% of the total diet weight, it was possible to increase the livestock survival rate by 2%, the hatching egg yield by 4.9%, and egg production by 1.46-1.11 PCs. Providing poultry with vitamin-rich green feed increased the content of carotenoids in egg yolk from 1.62 to 3.50 μg and that of Vitamins A and B_2_ by 3.19 and 2.32 μg, respectively, compared with the control group. Among all the investigated groups, the best indicators were found in experimental group 2, in which the use of feed nitrogen and the digestibility of protein and fat increased by, 2.6, 1.5 and 1.1% respectively. Furthermore, the lowest feed cost indicators per 10 eggs were observed in the same group. This study confirmed the economic effect, which amounted to 87.02 thousand rubles per 1000 animals. This fact emphasizes the beneficial use of hydroponic green herbage in the diets of parent flock geese.

## Authors’ Contributions

RG and DK supervised the study. RG, DK, CY, and SK conducted the study. DK and CY did the statistical analysis. MK and SK prepared the tables and figures. RG, DK, CY, and SK edited the manuscript. All authors read and approved the final manuscript.
